# Reduced graphene oxide: nanotoxicological profile in rats

**DOI:** 10.1186/s12951-016-0206-9

**Published:** 2016-06-24

**Authors:** Monique Culturato Padilha Mendonça, Edilene Siqueira Soares, Marcelo Bispo de Jesus, Helder José Ceragioli, Silvia Pierre Irazusta, Ângela Giovana Batista, Marco Aurélio Ramirez Vinolo, Mário Roberto Maróstica Júnior, Maria Alice da Cruz-Höfling

**Affiliations:** Department of Pharmacology, Faculty of Medical Sciences, State University of Campinas, Campinas, SP 13083-881 Brazil; Department of Biochemistry and Tissue Biology, Institute of Biology, State University of Campinas, Campinas, SP Brazil; Department of Semiconductors, Instruments and Photonics, School of Electrical and Computer Engineering, State University of Campinas, Campinas, SP Brazil; Faculty of Technology of Sorocaba, State Center of Paula Souza Technological Education, Sorocaba, SP Brazil; Department of Food and Nutrition, State University of Campinas, Campinas, SP Brazil; Department of Genetics, Evolution and Bioagents, Institute of Biology, State University of Campinas, Campinas, SP Brazil

**Keywords:** Nanoparticles, Blood–brain barrier, Toxicity

## Abstract

**Background:**

We have previously demonstrated that reduced graphene oxide (rGO) administered intravenously in rats was detected inside the hippocampus after downregulation of the tight and adherens junction proteins of the blood–brain barrier. While down-regulators of junctional proteins could be useful tools for drug delivery through the paracellular pathway, concerns over toxicity must be investigated before clinical application. Herein, our purpose was to trace whether the rGO inside the hippocampus triggered toxic alterations in this brain region and in target organs (blood, liver and kidney) of rats at various time points (15 min, 1, 3 h and 7 days).

**Results:**

The assessed rGO-treated rats (7 mg/kg) were clinically indistinguishable from controls at all the time points. Hematological, histopathological (neurons and astrocytes markers), biochemical (nephrotoxicity and hepatotoxicity assessment) and genotoxicological based tests showed that systemic rGO single injection seemed to produce minimal toxicological effects at the time points assessed. Relative to control, the only change was a decrease in the blood urea nitrogen level 3 h post-treatment and increases in superoxide dismutase activity 1 h and 7 days post-treatment. While no alteration in leukocyte parameters was detected between control and rGO-treated animals, time-dependent leukocytosis (rGO-1 h versus rGO-3 h) and leukopenia (rGO-3 h versus rGO-7 days) was observed intra-treated groups. Nevertheless, no inflammatory response was induced in serum and hippocampus at any time.

**Conclusions:**

The toxic effects seemed to be peripheral and transitory in the short-term analysis after systemic administration of rGO. The effects were self-limited and non-significant even at 7 days post-rGO administration.

## Background

Graphene is a single layer of densely packed, regular sp^2^-bonded carbon atoms arranged in a hexagonally two-dimensional structure [1]. Graphene and derivatives, such as graphene oxide (GO) and reduced graphene oxide (rGO), have attracted significant interest in many technological fields due to their unique electronic, optical, magnetic, thermal and mechanical properties. These properties have led to broad-spectrum material and biomedical applications, such as the use in biosensors, optical imaging, drug/gene delivery, photothermal therapy and tissue engineering [2–6].

With regard to rGO, the results of toxicological studies appear to be inconclusive, as there is no broad consensus on whether it is non-toxic and biocompatible [7–9]. These discrepancies have been attributed to many aspects related to the physicochemical properties (e.g., size, shape, surface chemistry, composition and aggregation) of the nanomaterial [10], and the experimental design used [11].

In our pioneer work with rGO, we demonstrated that rGO administered intravenously (i.v) through the tail vein of rats was further detected inside the brain tissue and particularly concentrated in the thalamus and hippocampus. We also found that Evans blue vital stain infusion in rGO-treated rats escaped from the peripheral circulation and entered the brain, indicating blood-brain barrier (BBB) disruption. With focus in hippocampus, we found that the possible entrance door of rGO into this brain region might have been in the course of a transient downregulation of the junctional proteins of the capillary endothelium [12]. The elaborate organization of such proteins maintains tightly attached to each other the capillary endothelial cells preventing the passage of substances through the paracellular pathway [13]. Such temporary decrease of the paracellular tightness of the BBB, the presence of rGO inside the hippocampus and the unnoticeable negative effect to animals’ exploratory behavior was predictor of a positive outcome in studies related to the toxicity of rGO.

In general, the toxicity of nanomaterials has been evaluated through their capacity to interfere in cellular mechanisms related to allergy, fibrosis, organ failure, hemocompatibility, neurotoxicity, nephrotoxicity and hepatotoxicity [14].

In the present study, we used the same experimental design and animal model as in our previous study [12] to delineate a possible toxic profile of rGO. The focus of the study was the hippocampus, and the peripheral organs (blood, kidney and liver) recognized as impact organs against xenobiotics. The purpose of the present study was to expand the understanding of rGO-tissue interactions in vivo, promoting safe and responsible use of rGO-based technology for future therapeutic application studies.

## Results and discussion

### rGO did not induce clinical signs of neurotoxicity in rats

A clinical evaluation was performed in rats before and after rGO injection in search of possible evidence of side-effects. Signs of tremor, piloerection, salivation, lacrimation, dyspnea, convulsions, hindlimb and forelimb grip strength, or other motor abnormalities were examined as symptoms of neurotoxic insult [15]. Animals from the rGO-treated groups did not show any of the signs described in Table 1 at any of the time-frame of the study (15 min, 1, 3 h and 7 days). The liveliness and exploratory behavior, typical of healthy rats, was equally observed in the control and rGO-treated animals.

**Table 1:** Summary of clinical signs and behavioral response destined to evaluate neurotoxicity before and after rGO i.v administration (7 mg/kg)

Autonomic nervous system	Peripheral nervous system (neuromuscular)	Behavioral (activity)	Central nervous system (excitability)
Lacrimation	Righting reflex	Motor activity	Clonic movements
Salivation	Hindlimb grip strength	Home cage posture	Tonic movements
Excretion	Forelimb grip strength	Rearing	
Piloerection			

All the parameters evaluated in rGO-treated rats showed no discrepancy relative to the observed in rats treated with vehicle (control)

n = 3–5 per time of sampling; n = 5 control group

Differently, Zhang and collaborators [16] reported that rGO nanosheets orally administered to mice caused a short-term decrease in locomotor activity and neuromuscular coordination. Other signs such anxiety-like, exploratory, or spatial learning and memory behaviors remained unnoticed. We suggest that the differences in relation to our findings are attributed do some aspects: (1) while we administered a single i.v. dose (7 mg/kg) to rats, Zhang et al. used high-dose (60 mg/kg administered orally for five consecutive days) to mice; (2) while our rGO was 342 ± 23.5 nm in size, theirs were small (87.97 ± 30.83) and large-sized (472.08 ± 249.17 nm). We conclude that experimental design, animal model and physicochemical characteristics of the nanomaterials have a key role in the development of toxic manifestations.

### rGO did not alter the neuronal viability marker and did not promote astrogliosis in the hippocampus of rats

Since rGO i.v. administration was shown to downregulate the junctional proteins responsible for the tight apposition of the endothelial cells of the BBB and was detected inside the brain [12], the next step was to investigate possible neurotoxic effects in the tissue. Firstly, we performed a histological evaluation of hematoxylin-eosin-stained hippocampus in search of tissue damage, inflammation or necrosis. Secondly, we evaluated the nuclear antigen protein (NeuN)—a marker of neuron maturation and viability—and the glial fibrillary acidic protein (GFAP), a protein of the intermediate filament of astrocytes and a marker of reactivity by triggering mechanical strength of the glia cytoskeleton in response to noxious stimuli to the brain. The relevancy of analyzing neurons and astrocytes rely on the fact that together with capillary endothelial cells and pericytes they constitute the neurovascular unit, a concept highlighting the functional cell–cell interactions which support BBB function [17, 18].

**Fig. 1:**
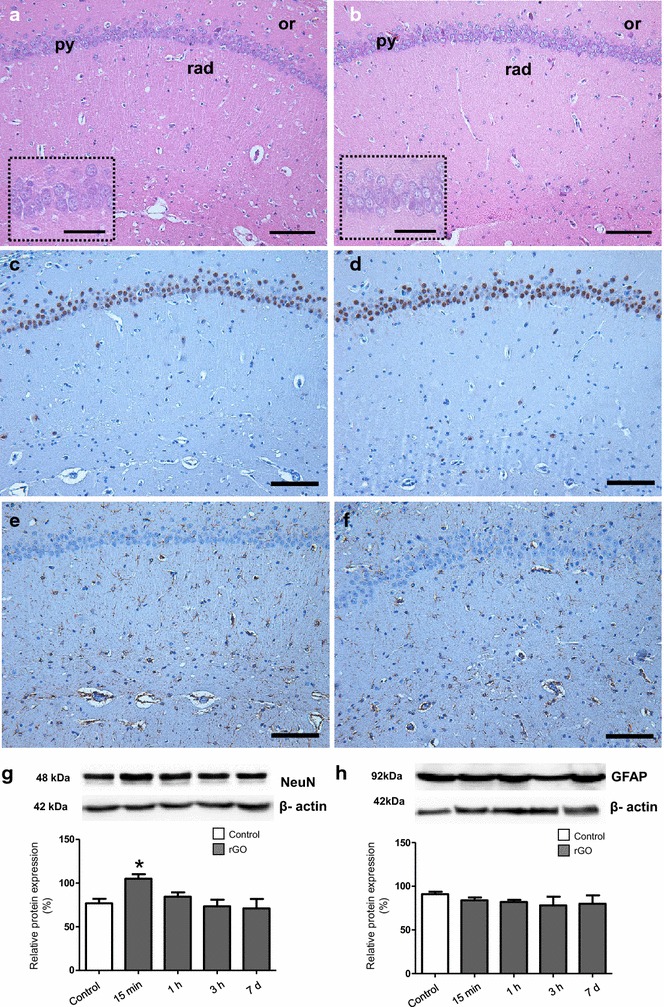
Light micrographs of CA1 hippocampal subfield of rats 1 h after i.v. injection of vehicle (**a**) and 15 min after i.v. injection of rGO (**b**), Hematoxylin-eosin. *Ins*ets show greater magnification of hippocampal pyramidal neurons with preserved euchromatic nuclei and visible nucleoli both in control and treated samples. Or *stratum oriens*; Py *stratum pyramidale*; Rad *stratum radiatum*. NeuN and GFAP labeling of 1 h-control rats (**c**, **e**) and 15 min after i.v. injection of rGO (**d**, **f**), respectively. Western blot signals of NeuN (**g**) and GFAP (**h**) after administration of rGO (7 mg/kg) in hippocampal tissue lysates were quantified densitometrically and normalized to an internal standard protein (β-actin). The results were shown as a percentage of control (100 %), and represent mean ± SEM (n = 5 rats/interval). *p < 0.05 indicates significant difference relative to control. One-way ANOVA, Bonferroni post hoc test; *Bars* 100 µm (**a**–**f**), 50 µm (*insets*)

The histological analysis of the hippocampus of treated animals showed that morphologically it did not differ from that observed in the control group regardless of the time following administration of rGO (Fig. 1a, b). Considering the neurons, NeuN immunolabeling showed that the nucleus of pyramidal neurons of the *Cornu ammonis* (CA) subfields (Fig. 1c, d) and the granule neurons of the dentate gyrus (not shown) of the hippocampus were typically reactive, not differing among treated and control animals. However, the NeuN content evaluated by immunoblotting in the hippocampal homogenates of rGO-treated animals (Fig. 1g) showed an immediate but episodic rise of 37 % (p < 0.05) in the protein expression level at 15 min. Thereafter, at 1, 3 h and 7 days NeuN immunolabeling and protein content returned to control basal levels. It is probable that a feeble mechanism of neurotoxicity had been immediately triggered after rGO injection, but it did not result in major change in the expression of NeuN. The increases in NeuN expression suggests enhanced or at least maintained neuronal viability. In addition, it means hippocampus plasticity, in which transitory migration of neurons may occur in response to local insult, resulting in fleeting increases of NeuN level.

With regard to the hippocampal astrocytes, a possible reaction against rGO would be by hypertrophy of the cytoskeleton and/or cell hyperplasia, a process known as reactive gliosis or astrogliosis [19]. Figure 1 illustrates GFAP labeling in the hippocampal CA1 region of a rat treated with a vehicle (panel E) and 15 min after rGO administration (panel F). Regardless of time post-rGO exposure, we observed soma and processes of the hippocampal astrocytes GFAP-positive, similar to that observed in control group. Likewise, the other hippocampal subfields, CA2, CA3, and dentate gyrus showed no difference between rGO-treated and control animals (data not shown). Quantitative analysis of the hippocampal homogenate by western blotting showed that treatment with rGO resulted in no significant alterations in GFAP levels over time, revealing absence of reactive astrogliosis (Fig. 1h). This indicates that rGO systemic injection did not induce substantial reactive response in astrocytes.

### rGO did not alter hematological parameters relative to control

In order to retain their engineered functions in vivo, nanomaterials used for biomedical applications should be compatible with blood [8]. No significant changes in any of the hematological parameters were observed in comparison with the control group. However, intra-group analysis revealed differences in the number of white blood cells (WBC) (Table 2). The rGO-3 h group showed a 59 % increase in the number of WBC relative to the rGO-15 min group, thus characterizing a time-dependent intra-rGO group leukocytosis (p < 0.05). This effect was transient as it was followed by a 115 % decrease when comparing the rGO-3 h group with the rGO-7 day group, hence characterizing an intra-rGO group time-dependent leukopenia (p < 0.01). All the other parameters showed steady figures over time with values at 7 days practically the same as those exhibited by control group.

**Table 2:** rGO effects on hematological parameters of male Wistar rats

Group	Unit	Control	rGO 15 min	rGO 1 h	rGO 3 h	rGO 7 days
RBC	10^6^/μl	7.32 ± 0.09	7.16 ± 0.17	7.45 ± 0.02	7.27 ± 0.11	7.48 ± 0.04
Hb	g/dl	14.27 ± 0.24	13.73 ± 0.12	14.37 ± 0.14	14.00 ± 0.24	14.32 ± 0.37
Hct	%	46.87 ± 0.52	46.13 ± 0.88	48.83 ± 0.57	47.58 ± 0.24	47.70 ± 0.40
MCV	fl	64.00 ± 0.60	64.60 ± 0.23	65.53 ± 1.02	65.44 ± 0.66	63.44 ± 0.49
MCH	pg	19.03 ± 0.23	19.03 ± 0.32	19.27 ± 0.24	19.22 ± 0.21	19.24 ± 0.26
MCHC	g/dl	29.80 ± 0.40	29.57 ± 0.40	29.43 ± 0.08	29.42 ± 0.42	30.38 ± 0.33
PLT	10^3^/μl	638.3 ± 5.84	650.0 ± 6.92	649.0 ± 10.69	624.2 ± 25.81	636.2 ± 59.19
WBC	10^3^/μl	7.63 ± 0.60	6.80 ± 1.41*	7.10 ± 0.30	10.83 ± 0.55*^,#^	5.03 ± 0.56^#^

The same symbol in each column indicates a significant difference between groups

One-way ANOVA, Bonferroni post hoc test; data were shown as mean ± SEM, n = 3–5 in each group

*RBC* red blood cell; *Hb* hemoglobin; *Hct* hematocrit; *MCV* mean corpuscular volume; *MCH* mean corpuscular hemoglobin; *MCHC* mean corpuscular hemoglobin concentration; *PLT* platelet; *WBC* white blood cell

*p < 0.05; ^#^p < 0.01

It has been demonstrated that graphene-based nanomaterials were compatible with blood and did not cause hemolysis, platelet activation and changes in coagulation or abnormalities in hematological parameters [20]. In contrast, Singh et al. [21] reported that intravenous administration of GO (250 μg/kg body weight) caused strong platelet aggregation and extensive thromboembolism in mice. Interestingly, much less platelet aggregation occurred with rGO, which may be correlated to difference in surface charge distribution from GO as the same concentration and animal model were used.

In rodents, several types of stimulus such as exercise, environmental factors, nutrition (food and water) and inflammatory response [22], can influence WBC levels [23]. In comparison with the control group, our results showed that rGO-treated animals did not alter significantly leukocytes number. The variability seen among rGO groups on WBC counts might be considered rather moderate, as the values observed here are still within the reference ranges found in rats [23, 24].

**Fig. 2:**
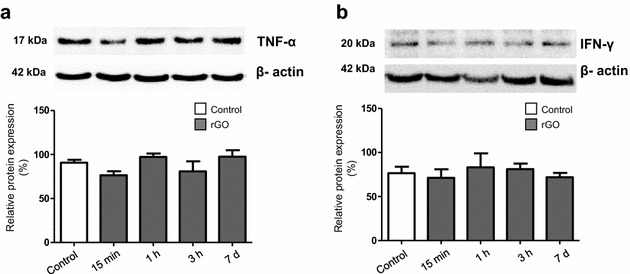
Expression of TNF-α (**a**) and IFN-γ (**b**) in hippocampal homogenates after rGO i.v. administration (7 mg/kg). Western blot signals were densitometrically quantified, normalized to an internal standard (β-actin) and the results expressed as percentage of control (100 %). One-way ANOVA, Bonferroni post hoc test; data were shown as mean ±SEM; n = 5/group

To confirm that the time-dependent leukocytosis (from 15 min to 3 h) did not represent an inflammatory reaction in response to rGO we assessed the levels of interleukin 6 (IL-6) and tumor necrosis factor-α (TNF-α) pro-inflammatory cytokines in serum (ELISA assay) and the expression of TNF-α and interferon-γ (IFN-γ) in the hippocampal homogenates (WB data). IL-6 and TNF-α was undetectable in the serum of rGO-treated rats. All samples measured less than the lowest rat standard levels, 62.5 pg/mL for IL-6 and 125 pg/ml for TNF-α. Likewise, no significant change was observed in the expression of either inflammatory marker over time (Fig. 2). Based on these results and in the absence of inflammatory cell infiltration (Fig. 1b), it appears that the increase in the number of WBC cells intra-rGO-treated groups cannot be associated to any inflammatory process.

### Morphological and functional evaluations show minimal effects of rGO in liver and kidney

To better analyze the safety of using rGO, hepatotoxicity and nephrotoxicity were evaluated to determine whether or not rGO caused alterations in liver and kidney morphology and function. These organs are important components for detoxification and clearance of nanoparticles, respectively [25].

**Fig. 3:**
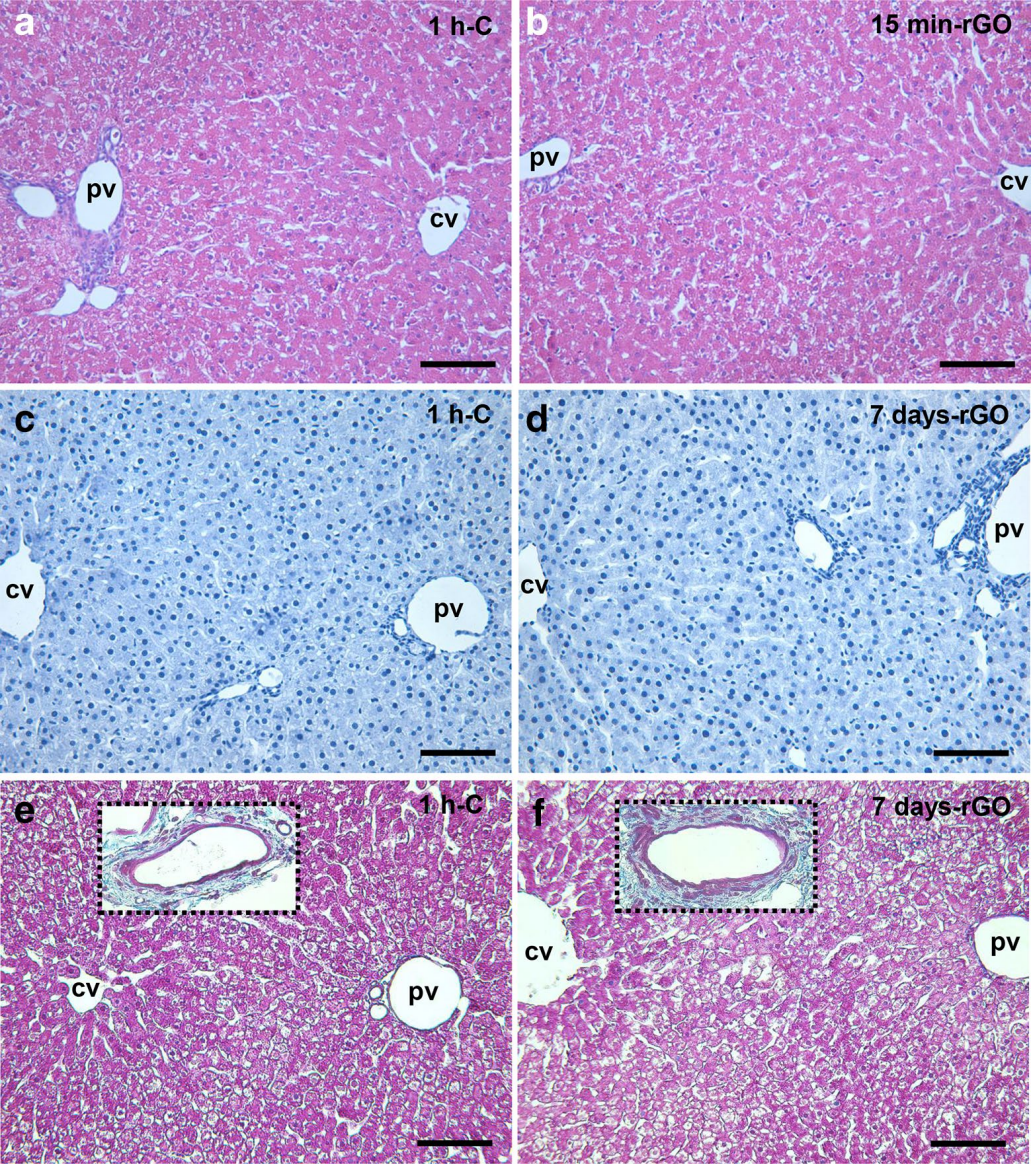
Light micrographs of parts of hepatic lobules at 1 h-control (**a**, **c**, **e**), rGO-15 min (**b**) and 7 days—rGO treated rats (**d**, **f**). Representative photomicrographs of the hepatic parenchyma stained with Hematoxylin-eosin (**a**, **b**); treated for immunostaining of anti-caspase 9 (**c**, **d**) and stained with Masson’s trichrome staining for detecting collagen type 1 in normal (**e**) and rGO-treated (**f**) tissue. *Insets* depict collagen fibers around the blood vessels. *pv* portal vein; *cv* central vein. *Bars* 100 µm

rGO systemic administration did not produce detectable changes in the lobular architecture of the liver, which was preserved and remained normal in all treated rats at the studied time-frame (Fig. 3a, b) even 7 days after rGO administration (data not shown). Likewise, no immuno-positive apoptotic cells were detected (Fig. 3c, d). Typically, collagen fiber density was only visible in the hepatic portal venous system and this was unchanged in control and rGO-treated tissue (insets Fig. 3e, f); equally no alterations in the connective tissue were detectable in the peri-sinusoidal space in rGO-treated samples stained with Masson’s trichrome (Fig. 3f) compared to control (Fig. 3e). The data suggest that in these experimental conditions there were absence of hepatocytes apoptosis and tissue fibrosis.

**Fig. 4:**
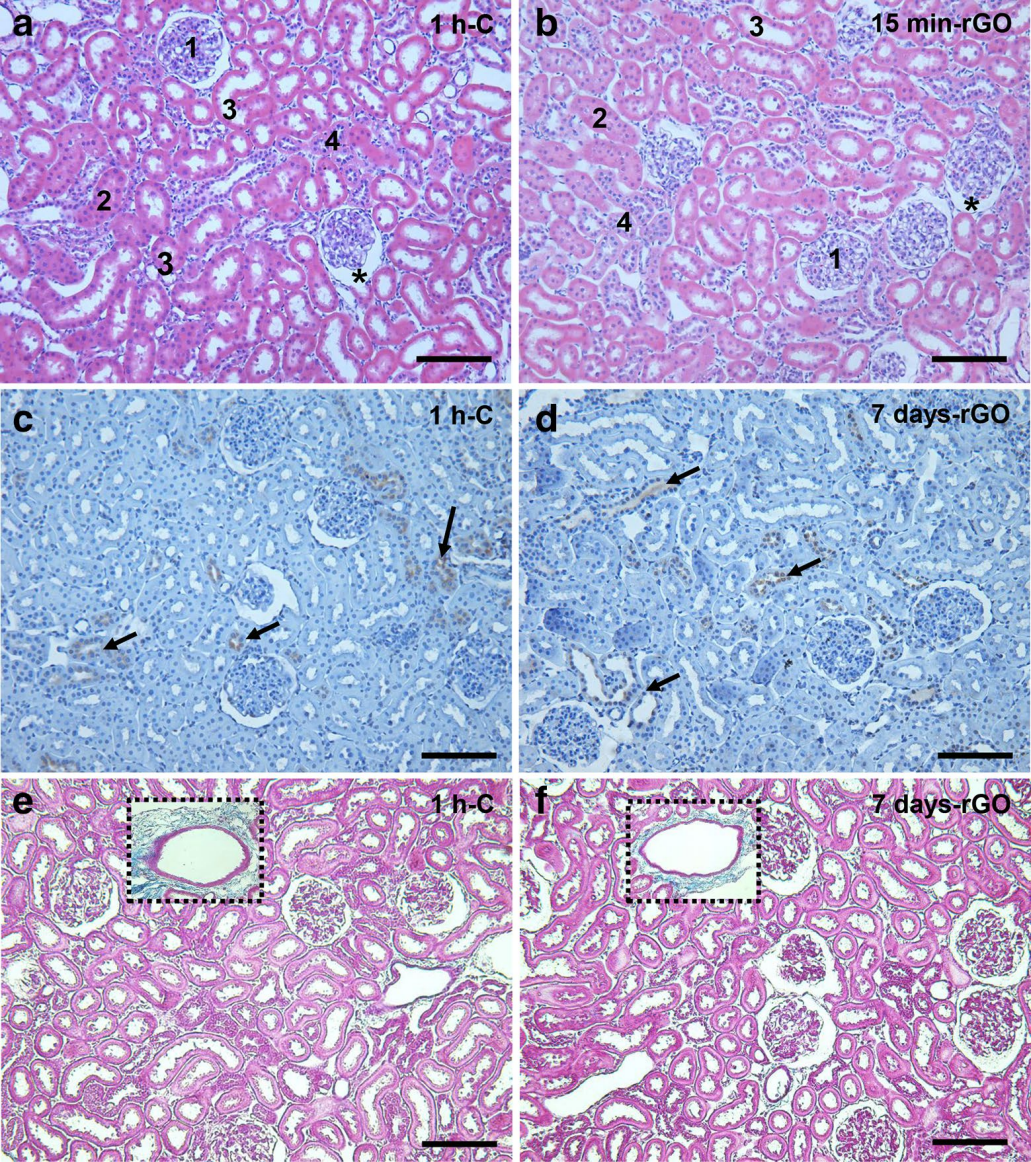
Renal cortical sections stained with Hematoxylin-eosin (**a**, **b**), immunostained for caspase 9 (**c**, **d**) and stained with Masson’s trichrome (**e**, **f**). The sections were obtained from vehicle-treated (control—1 h) rats (**a**, **c**, **e**) and 15 min (**b**) and 7 days (**d**, **f**) after intravenous injection of rGO. Note that there is no change between the aspect of control and rGO-treated sections in each of the staining assessments. The widening of the tubular lumen and capsular space was observed in control and rGO samples due to perfusion fixation. *Insets* depict that collagen density (just in large blood veins of renal stroma) was the same for control and rats treated with rGO. *1* Renal glomeruli; *2* proximal tubule; *3* distal tubule; *4* collecting tubule; * Bowman’s space of the renal corpuscle. *Arrows* point to the immuno-positive apoptotic cells (*brown*). *Bars* 100 µm for all *panels*

With regard to kidneys, the tubular, glomerular tufts and renal corpuscles and interstitium of rGO-treated rats were normal in appearance and did not differ from control (Fig. 4a, b). Caspase-9 immunostaining allowed identifying dispersed nephron segments with apoptotic cells (brown-labeled cells) both in control (Fig. 4c) and rGO-treated rats (Fig. 4d). As is common, the renal parenchyma shows minimal connective tissue support in physiologic condition (Fig. 4e), and no alteration was apparent in rGO-treated animals (Fig. 4h).

Additionally, we evaluated the levels of aminotransferases, alanine aminotransferase (ALT) and aspartate aminotransferase (AST), in serum, as in drug safety studies these enzymes are key indicators of drug-induced liver toxicity involving laboratory animals and patients. These enzymes are abundant within hepatocytes and catalyze the formation of glutamate through the transfer of an amino group [26]. Thus, increased levels of these enzymes in the blood are one of the first laboratory signs of hepatic dysfunction. As shown in Table 3, no alteration was found in the enzyme levels in comparison with control over the time-frame scheduled.

**Table 3:** Effects of rGO on biochemical parameters of hepatic and renal function

	Unit	Control	rGO 15 min	rGO 1 h	rGO 3 h	rGO 7 days
ALT	U/L	44 ± 3.5	56 ± 3.9	54 ± 0.5	44 ± 3.2	48 ± 2.3
AST	U/L	148 ± 9.9	177 ± 8.4	152 ± 7.5	156 ± 9.5	151 ± 6.6
BUN	mg/dL	48 ± 2.2^#^	57 ± 3.2*^,∇^	45 ± 3.3*	35 ± 0.7^#,∇^	48 ± 1.8
Creatinine	mg/dL	0.31 ± 0.003	0.35 ± 0.017	0.29 ± 0.021	0.30 ± 0.017	0.34 ± 0.020

Values are mean ± SEM of 3–5 animals in each group. The same symbol in each column indicates a significant difference between groups

One-way ANOVA, Bonferroni post hoc test

*ALT* alanine aminotransferase; *AST* aspartate aminotransferase; *BUN* blood urea nitrogen

^#,^* p < 0.05; ^∇^p < 0.001

Next, renal glomerular function was estimated by assessing creatinine and blood urea nitrogen (BUN) levels, two markers of renal function [27]. We did not observe alterations in creatinine levels; but found a transitory and unexpected 37 % decrease in BUN levels at 3 h in the rGO-treated animals, compared with the control group (p < 0.05, Table 3). Decreases in BUN level were also found as time post-rGO exposure advanced: 27 % (p < 0.05) decrease in rGO-15 min versus rGO-1 h and 63 % (p < 0.001) decrease in rGO-15 min versus rGO-3 h. One week after rGO treatment, BUN levels had returned to the control rate, which may indicate that in case of hepatotoxicity or nephrotoxicity occurrence they were short-lived. Moreover, the histopathological examination of kidney and liver morphology showed none abnormality in the rGO-treated group.

BUN analysis also provides information about hepatic functioning, as the nitrogen from ammonia produced by the liver will participate in urea formation which will be further excreted by the kidney as a waste metabolism product. Increases in BUN levels indicate that either the kidney or the liver may not be functioning properly. In humans, low levels of BUN have been observed in several morbid conditions such as those caused by trauma, surgery, malnutrition and opioids and anabolic steroid use and fluid excess [27, 28], whereas low levels are not common and not usually reported in animals.

### Evaluation of oxidative stress and DNA damage generated by rGO

As one of the most important mechanisms explored in graphene-based nanomaterials studies, oxidative stress has been the focus of toxicological studies [9, 29]. It is well known that the generation and elimination of reactive oxygen species (ROS) is dynamically balanced inside cells, and severe increases in ROS levels may induce genotoxicity, protein inactivation, lipid peroxidation, mitochondrial dysfunction, and eventually cell death by apoptosis or necrosis [30–32].

Herein, the influence of rGO in the response of biomarkers of oxidative stress was assessed through the expression and activities of the antioxidant enzymes superoxide dismutase (SOD) and catalase (CAT), known to be involved in the detoxification of hydrogen peroxide (H_2_O_2_). SOD converts the superoxide radical into H_2_O_2_ while CAT converts H_2_O_2_ into water [33]. We further evaluated the thiobarbituric acid reactive substances (TBARS), as a marker of lipid peroxidation.

rGO systemic single injection induced upregulation in serum SOD activity, while CAT activity was unaffected. Likewise, no significant differences were detected in serum TBARS levels between rGO-treated and control animals, indicating that lipid peroxidation was unaltered (Table 4). SOD activity rose progressively from 15 min up to 7 days. Relative to control, SOD activity increased at 1 h (54 %, p < 0.05) and at 7 days (69 %, p < 0.01), after the injection of rGO. Intra-group analysis revealed a significant increase (57 %, p < 0.01) in rGO-7 days relative to rGO-15 min.

**Table 4:** rGO effects on serum SOD, catalase and TBARS levels in male Wistar rats

	Unit	Control	rGO 15 min	rGO 1 h	rGO 3 h	rGO 7 days
SOD	U/ml	13 ± 0.4^#,^*	14 ± 0.8^∇^	20 ± 0.6^#^	20 ± 2.6	22 ± 1.1*^,∇^
CAT	nmol/min/ml	129 ± 2.8	151 ± 12.5	112 ± 28.8	88 ± 17.3	120 ± 6.1
TBARS	nmol/ml	7.6 ± 1.0	7.7 ± 1.7	7.4 ± 2.4	6.2 ± 0.3	5.7 ± 1.3

Values are mean ± SEM of 3–4 animals in each group. The same symbol in each column indicates a significant difference between groups

One-way ANOVA, Bonferroni post hoc test

*SOD* superoxide dismutase; *CAT* catalase; *TBARS* thiobarbituric acid reactive substances

^#^p < 0.05; *^,∇^p < 0.01

**Fig. 5:**
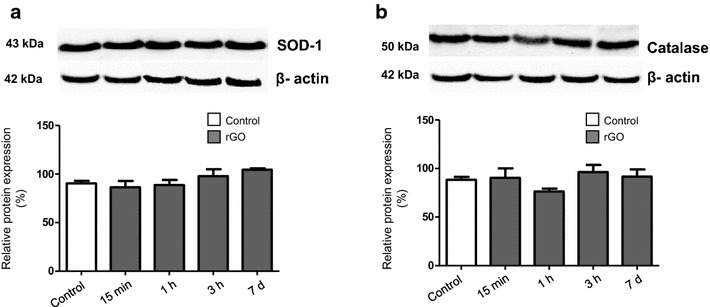
Expression of SOD-1 (**a**) and catalase (**b**) in hippocampal homogenates after rGO i.v. administration (7 mg/kg). Western blot signals were densitometrically quantified, normalized to an internal standard (β-actin) and the results expressed as percentage of control (100 %). One-way ANOVA, Bonferroni post hoc test; data were shown as mean ± SEM; n = 5 in each group

Western blot analyses did not show significant difference in SOD-1 or CAT protein expression over the time course analyzed (Fig. 5a, b), suggesting that rGO did not alter transduction signal.

Therefore, we decided to investigate whether the generation of oxidant compounds by systemic rGO injection, as inferred by the increase in the SOD activity, had induced genotoxicity and cell death under the influence of rGO.

As shown in Table 5, rGO did not induce cytogenetic damage. There was no difference in the frequency of micronucleated erythrocytes in the circulating blood of control rats and in the blood collected from rats 7 days after a single i.v. injection of rGO. In human glioblastoma cell lines U87 and U118, GO and rGO decreased cell viability and proliferation, however rGO was more toxic than GO. In rGO-treated U87 tumors the expression of caspase-3 was 96 % higher compared with controls [34]. Herein, no substantial differences were observed in caspase 3 levels in the hippocampal homogenates between controls and animals treated with rGO.

**Table 5:** Effect of rGO on the frequency of polychromatic erythrocytes with micronuclei in the peripheral blood and caspase-3 expression

	Control	rGO 15 min	rGO 1 h	rGO 3 h	rGO 7 days
Micronucleus index	0.68 ± 0.13	NA	NA	NA	0.64 ± 0.19
Caspase 3	92.46 ± 2.88	101.3 ± 11.66	91.71 ± 8.00	89.05 ± 15.99	95.97 ± 14.46

Protein expression levels were expressed as percentage of control (100 %) after normalization to an internal standard (β-actin). One-way ANOVA, Bonferroni post hoc test; data were shown as mean ± SEM; n = 5 in each group

*NA* not assessed

We suggest that induction of oxidative stress by rGO was moderate and that the reductive defense system provided by SOD activity was triggered to restore the oxidant/antioxidant balance.

## Conclusions

Overall, the intravenous administration of rGO (7 mg/kg single dose) led to minor signs of toxicity in the blood, liver and kidney after 7 days with no sign of inflammation process in course. These effects were transitory and did not lead to permanent damage. Also, no perceptible change was observed in hippocampal neurons or astrocytes response, despite a previous study demonstrating BBB disruption and detecting rGO distribution inside this brain region [12]. The activity of control and rGO-treated animals in their cages was the same, with no apparent clinical signs of toxic manifestation. In conclusion, the data suggests that systemic administration offers no significant health risk for rats in these experimental conditions. Nevertheless, it seems clear that the interactions between graphene-based materials with biological systems are highly dependent on the experimental design used and the physicochemical properties of the nanomaterial. For the development of graphene-based nanomedicine, it is important the development of systematic toxicological investigations to fully understand the biological effects and address safety concerns before the practical application of any graphene-based materials in the clinic. The present study provides a basis for further toxicological studies of rGO after long-term in vivo exposure, and is an incentive for studies of the mechanisms underlying rGO and cell/biological system interaction.

## Methods

### Preparation and characterization of rGO

The processing for rGO was the same described before to maintain identical properties as the used in our previous study [12]. Briefly, rGO was prepared after catalytic conversion using a copper substratum to which was added 1 ml of polyaniline diluted in dimethylformamide (Synth, São Paulo, SP, Brazil), after which it was allowed drying for 2 h at room temperature. After, 0.2 ml of nickel nitrate dissolved in pure acetone (Synth) was added to the preparation which was subsequently placed within a chemical vapor deposition reactor assisted by a hot filament. The hydrocarbons used as a carbon source were camphor and acetone. Raman spectrometry (Renishaw, Wotton-under-Edge, UK) for molecular structure characterization, Field Emission Scanning Electron Microscopy (JEOl JSM-6330F, Japan) for morphological description and dynamic light scattering for measurement of size, zeta potential and polydispersity index (PDI) (ZetaPALS Zeta Potential Analyzer, Brookhaven Instruments, NY, USA) confirmed that rGO characteristics were the same exhibited in our previous study (sized 342 ± 23.5 nm, −25 ± 0.18 mV zeta potential, 0.56 ± 0.03 PDI).

**Fig. 6:**
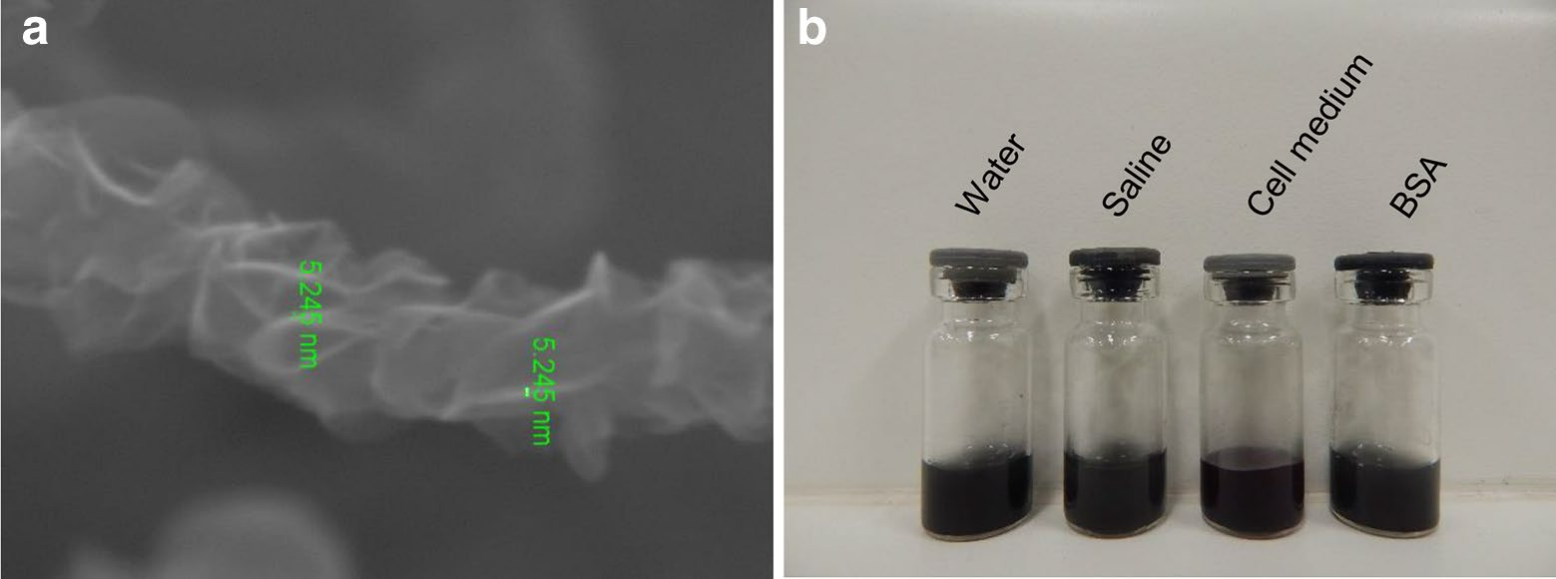
FE-SEM image of rGO sheet with a thickness of ~5 nm (**a**). rGO suspension in water, 0.9 % saline, DMEM cell medium and BSA displaying great stability (**b**)

In this work, we performed additional information about physicochemical properties of rGO. The average thickness of the rGO sheet measured by Field Emission Scanning Electron Microscope (FE-SEM) (Zeiss Supra 55 VP-SEM) was estimated to be ∼5 nm (Fig. 6a). rGO showed remarkable stability in water and different physiological solutions including 0.9 % saline solution, Dulbecco’s Modified Eagle Medium (DMEM), and bovine serum albumin (BSA) (Fig. 6b).

### Animal treatment

Experiments were carried out in accordance with the Brazilian Society of Laboratory Animal Science guidelines and approved by the Institutional Committee for Ethics in Animal Use (CEUA/IB/UNICAMP, protocol n. 2884-1). The experimental animals used in our previous study [12] were the same used in the present one. We used healthy male Wistar rats (average weight 180–220 g; 6 weeks old; *n* = 3–5 in each group) for evaluating the nanotoxicity of rGO.

Animals (*Rattus norvegicus*) received a single tail vein injection of rGO (7 mg/kg dose; concentration of 1 mg*/*ml) [35], while the control group was given the same volume of vehicle (sterile distilled water). Fifteen minutes, 1, 3 h and 7 days after the i.v. administration of rGO, the animals were euthanized by carbon dioxide (CO_2_) inhalation (western blotting analysis) or anesthetics overdose [3:1 mixture of ketamine chloride (Dopalen^®^, 100 mg/kg body weight, Fortvale, Valinhos, SP, Brazil) and xylazine chloride (Anasedan^®^, 10 mg/kg body weight, Fortvale)] and the target samples (blood, brain, liver and kidneys) immediately removed. Animals of the control group received an i.v. injection of vehicle and were euthanized 1 h later. A single control group was used as preliminary experiments showed no time difference relative to data.

### Neurotoxicity evaluation

The clinical and behavioral signs were evaluated in control and rGO-treated rats using the functional observation battery described by Moser [36]. All observations were performed by the same trained observer who was unaware of treatments.

### Systemic toxicological profile of rGO

Fifteen minutes, 1, 3 h and 7 days after treatment with rGO, the animals were deeply anesthetized and blood samples were collected via cardiac puncture immediately prior to transcardial perfusion and divided into two parts, (1) collected into EDTA-containing tubes and used for hematological and genotoxicity studies, and (2) collected into serum separator gel tubes and used for enzyme-linked immunosorbent assay (ELISA), biochemical studies and analysis of the activity of antioxidant enzymes.

#### Hematological studies

Hematological parameters—numbers of red blood cells, hemoglobin, hematocrit, mean cell volume, mean corpuscular hemoglobin, mean corpuscular hemoglobin concentration, platelet and white blood cell count—were determined using an automated hematology analyzer (Coulter T540 hematology system; Fullerton, CA, USA).

#### ELISA

Blood samples were centrifuged for 10 min at 3000 rpm and the resulting serum was stored at −80 °C. The cytokines IL-6 and of TNF-α were determined using Rat DuoSet ELISA kits (R&D Systems; Minneapolis, MN, USA) following the instructions supplied by the manufacturer. The limit of detection of these kits was 125 pg/ml for IL-6 and 62.5 pg/ml for TNF-α.

#### Biochemical parameters

The blood samples for biochemical analyses were centrifuged at 3000 rpm for 10 min, the serum aspirated and analyzed by an automated analyzer Cobas^®^ 6000 (Roche Diagnostics, Mannheim, Germany) for the determination of AST and ALT activities and the concentration of BUN and creatinine.

#### Antioxidant enzymes activity and lipid peroxidation evaluation

Lipid peroxidation was evaluated using TBARS technique described by Ohkawa, Ohishi, and Yagi [37] and adapted by Batista et al. [38], in which malondialdehyde and the final products of lipid peroxidation react with thiobarbituric acid, forming a pink-colored complex.

The enzyme antioxidant systems (SOD and CAT) in the serum samples were measured using colorimetric methods. The SOD activity in serum was obtained after reaction with hypoxanthine, nitroblue tetrazolium and 0.07 U of xanthine oxidase as described in detail before [38].

The CAT activity method was carried out based on the reaction of the enzyme with methanol and H_2_O_2_. Purpald (4-amino-3-hydrazino-5-mercapto-1,2,4-triazole) was used as chromogen and the resultant formaldehyde products was measured at 540 nm (adapted from [39]).

#### Genotoxicity assay

The genotoxic potential of rGO was evaluated by micronuclei assay in peripheral blood. The assay was performed in accordance with the Redbook 2000: IV.C.1.d Mammalian Erythrocyte Micronucleus Test [40]. Briefly, after fixation in methanol for 10 min, three good-quality smears prepared from each blood sample (n = 3 blood samples; total n = 9 smear slide/time) were left to air-dry, stained with Leishman solution for 12–15 min and then analyzed using an Olympus BX51 photomicroscope (Japan) at 400× or 1000× magnification, as required.

From each smear slide, the frequency of micronuclei were determined by counting a total of at least 1000 erythrocytes and expressed as per 1000 cells (%). Since the occurrence of possible mutagenesis was not observable at earlier periods of times (15 min, 1 and 3 h), the analysis was solely performed in the 7 day-samples.

#### Histopathological and immunohistochemistry assessment

Immediately after blood collection, the rats were perfused with physiological saline followed by 4 % paraformaldehyde in 0.1 M PBS, pH 7.4. The brains and peripheral organs (liver and kidney) of rGO-injected rats and controls were removed and routinely processed for paraffin embedding [41]. Coronal 5 μm thick sections of brain, liver and kidney were stained with Hematoxylin-eosin for histological evaluation, Masson’s trichrome technique to visualize collagen fibers or immunostained using primary antibodies (see Table 6) using standard protocols. Briefly, the endogenous peroxidase activity was quenched with 3 % hydrogen peroxide, (two cycles of 10 min) and epitope retrieval was accomplished with 10 mM sodium citrate buffer, pH 6.0, in a steamer (95–99 °C) for 30 min. Non-specific antigen binding was blocked with 5 % skimmed milk powder for 1 h. Slides were incubated with the primaries antibodies for 16–18 h in a humidified chamber at 4 °C. After washing twice with phosphate-buffered saline (PBS, pH 7.4), the slides were incubated with biotinylated anti-rabbit secondary antibody (EnVision_HRP link, Dako Cytomation, CA, USA) for 30 min at room temperature. Color was developed with a diaminobenzidine chromogenic solution (DAB+, Dako Cytomation) and nuclei were counterstained with Harry’s hematoxylin; after ethanol dehydration slides were mounted in Canada balsam. Negative control was done by replacing the primary antibody with 1 % PBS-bovine serum albumin. Images were captured on an Olympus BX51 photomicroscope (Japan).

**Table 6:** Primary antibodies used in this study

Antibody	Dilution	Supplier	Reference	Application
Caspase 3	1:400	Santa Cruz	Sc-7148	WB
Caspase 9	1:50	Sigma Aldrich	C7729	IHC
Catalase	1:400	Santa Cruz	sc-271242	WB
GFAP	1:100	Dako	Z0334	IHC
GFAP	1:500	Dako	Z0334	WB
IFN-γ	1:500	Santa Cruz	sc-9344	WB
NeuN	1:500	Merck Millipore	ABN78	IHC
NeuN	1:1000	Merck Millipore	ABN78	WB
SOD-1	1:500	Santa Cruz	sc-11407	WB
TNF-α	1:1000	Cell signaling	#11948	WB
β-Actin	1:1000	Sigma Aldrich	A2228	WB

#### Western blotting

Western blotting was performed in hippocampal homogenates (n = 5 for each time, including single control) as previously described [12]. After electrotransfer, the membranes were incubated with 5 % skimmed milk powder to block non-specific sites prior followed by washing with TBS-T (0.1 % Tris-buffered saline with 0.05 % Tween 20, pH 7.4). Subsequently, the membranes were incubated with primary antibodies (see Table 6). Then, the membrane were washed with TBS-T and incubated with HRP-labeled anti-mouse (for anti-catalase and anti-β-actin), anti-goat (for anti-IFN-γ) or anti-rabbit (anti-caspase 3, SOD-1, anti-GFAP, anti-NeuN and anti-TNF-α) secondary antibody (1:1000, Sigma Aldrich). Bands were visualized using a chemiluminescence kit (Super Signal West Pico Chemiluminescent Substrate; Pierce Biotechnology, Rockford, IL, USA) according to the manufacturer’s instructions. The luminescent signal from bands was captured by a G:BoxiChemi camera (Syngene, Cambridge, UK) and band intensities were quantified using Image J 1.45S (NIH, Bethesda, MD, USA). Blots were stripped and reprobed for β-actin to monitor protein loading the efficiency of blot transfer, and non-specific changes in protein levels.

### Statistical analysis

One-way ANOVA followed by Bonferroni post hoc test was performed for multiple variant analyses. Differences were considered statistically significant at p < 0.05. All values were expressed as the mean ± standard error of the mean (SEM). All analyses were done using Prism software, version 5 (GraphPad Inc., La Jolla, CA, USA).

## Abbreviations

ALT: alanine aminotransferase
AST: aspartate aminotransferase
BBB: blood–brain barrier
BUN: blood urea nitrogen
CA: *cornu ammonis*
CAT: catalase
ELISA: enzyme-linked immunosorbent assay
GFAP: glial fibrillary acidic protein
GO: graphene oxide
Hb: hemoglobin
Hct: hematocrit
IFN-γ: interferon-γ
IL-6: interleukin-6
MCH: mean corpuscular hemoglobin
MCHC: mean corpuscular hemoglobin concentration
MCV: mean corpuscular volume
NeuN: nuclear antigen protein
PDI: polydispersity index
PLT: platelet
RBC: red blood cell
rGO: reduced graphene oxide
ROS: reactive oxygen species
SOD: superoxide dismutase
TBARS: thiobarbituric acid reactive substances
TNF-α: tumor necrosis factor-α
WBC: white blood cell
